# Some Disturbances Caused by Diseases of the Teeth

**Published:** 1895-05

**Authors:** Edgar F. Stevens

**Affiliations:** Medford, Mass.


### SOME DISTURBANCES CAUSED BY DISEASES OF THE TEETH.[1]

BY EDGAR F. STEVENS, D.M.D., MEDFORD, MASS.

It is well known that few physicians, especially those who studied medicine twenty or more years ago, think of the teeth in connection with nervous disturbances of the body, the reason being that they have but a limited opportunity to learn anything connected with the teeth and oral cavity while in the medical school. But the time is coming when the student of medicine will have the same privilege of studying dental pathology that he now has of studying diseases of the eye, ear, throat, etc. Already one of our members holds the professorship of Dental Pathology in a Chicago Medical School, and I hope before long to see a graduate of our dental school lecturing on Dental Pathology to the medical student of Harvard. That the teeth are the cause of a great many disturbances not only of the parts contiguous to them, but often of parts far removed, is a fact which is recognized by us all, and it is not surprising when we bear in mind the intimate connection established between the teeth and other parts of the body by nerves, blood-vessels, and muscular tissue.

It is a wonder we have so little disturbance from some conditions. In the extraction of the teeth, for instance, the muscular and nerve fibres are broken, and the bony tissue split and slivered, yet, as a rule, how little attention or medication is needed. If an injury to any other part of the body were as extensive, it would produce a much greater amount of distress. We have all seen patients with high pulse and temperature, and in an extremely nervous condition caused by alveolar abscess. We are all familiar with the different phases of neuralgia caused by deposits of salivary calculus, exposure of pulp, calcification of pulp, pressure of gas, pressure of fillings on pulps, extended eruption, spiculæ or nodules of bone which have not been absorbed after extraction, and by many other causes. Under some circumstances the teeth and reproductive organs in females are in sympathetic discord; and cases are not wanting to prove that the dentist is capable of alleviating the distracting pains when the general practitioner is at a loss as to cause and treatment.

---

[1] Read before the Harvard Odontological Society, Boston, September 27, 1894.

I shall present to you to-night the record of a few cases of disturbances caused by dental irritation, which have come into my hands.

The first case is that of a lady about thirty years of age, who had supraorbital neuralgia. The pain would start a day or two before the menstrual flow in a second superior right molar, and shoot to the supraorbital region. It was intense, sharp, gnawing in character, and would generally last until the second day of the flow, when it would gradually pass away. She had been under treatment for neuralgia, but relief was only temporary. Through one of my patients she was induced to come to me for examination. The tooth in question looked all right, no cavities, no discoloration, not tender to touch, but from the nature of the pain which, as I said before, was of a sharp, gnawing character, I concluded that the pulp was calcified. I removed a large corono-mesial filling, which was in perfect condition, and cut down to the pulp-chamber without giving much pain, and found the body of the pulp entirely calcified, filling the entire chamber, but not attached to its walls. The root-canals were imperfectly filled with calcified tissue a third of their length. Touching the living pulp in the lower part of the canals would send a sharp pain to the supraorbital region. The patient remarked that "it was like taking a shock of electricity." The pulp-tissue was devitalized and removed, the canals filled, and the crown filling replaced. The neuralgia disappeared.

The second case is that of a lady pregnant for the first time, who, two weeks before the expected birth, suffered greatly from pain which started in an inferior molar, and was transferred to the uterus, then back to the tooth, and thus it continued changing from two o'clock in the morning till her physician arrived at eight o'clock, when he examined thoroughly and was positive the pains were not caused by the contraction of the uterus, and after giving an opiate called on me and asked if I thought the tooth had anything to do with the pain. I replied it was not improbable, and that the patient had better come to my office if she were able to do so. Upon her arrival I examined the mouth thoroughly. Her teeth were in an excellent condition, with the exception of the tooth in which the pain started. This tooth had large approximal alloy fillings, which were apparently doing good service, but the tooth was discolored and a trifle tender to the touch. Being satisfied the tooth had either a dead or a dying pulp, I opened into the pulp-chamber and found the pulp dead. I washed out the canals with peroxide of hydrogen, and applied a capsicum plaster to the gum, placing a pledget of cotton loosely in the cavity to keep out food.

The patient, who had been crying with the pain, which seemed to be intense in the uterus, but not very much so in the tooth, dried her eyes and said she was relieved. She had no recurrence of pain, and at the proper time gave birth to a healthy child. The tooth was afterwards treated and filled.

The third case is that of a man whom I found waiting in a carriage in front of my office one morning holding on to his head and acting very peculiarly. He followed me into the office and cried out in agony, "Doctor do something quickly for me, I am nearly crazy; it seems as if my head would split." I asked him if he hadn't better see a physician. He replied that he had seen one, and had been sent by him to me. I found by questioning him that the pain had started about a week previously in a second superior molar, and had not been very severe until the night before I saw him, when it began to "sing," as he termed it, and his whole head was in an intense neuralgic condition. I found a large alloy filling in the masticating surface, which I removed. The pulp-chamber was filled with red gutta-percha, also the entrances to the canals. With a stiff broach I found I could push through the gutta-percha in the buccal canals and touch nerve-tissue. The palatine canal was filled to the apex with the gutta-percha. I removed all the gutta-percha from the canals, and tried to obtund the pulp with cocaine (ten-per-cent. solution), but to no purpose, and decided as pain was so severe to etherize the patient and make short work of it. After etherizing and breaking up connection with a barbed broach the patient regained consciousness, and smilingly said the pain was entirely gone. At a subsequent day the canals were treated and filled.

The fourth case is that of a boy, eight years of age, who while riding on the rear platform of an electric car was struck by the brake-handle. In turning the corner the trolley slipped off the wire, the rope which is attached at one end to the trolley pole was, as is usual, fastened at the other end to the brake-handle, which was thrown round with such force that it struck the boy on the side of the lower jaw and knocked him against the door, bruising his shoulder quite badly. Five days after this occurred his physician brought him to me for examination. I found upon opening his mouth that the second inferior left deciduous molar had been recently removed (it was knocked out by the brake-handle), the first diciduous molar was displaced, the masticating surface presenting to the tongue, the roots projecting through the gum externally. There was also an incomplete fracture of the outer alveolar plate extending from the inferior canine to the sixth-year molar; a severe contusion of the left cheek and laceration of the mucous membrane, with a discharge of pus internally from a lacerated opening in the cheek. The roots of the displaced molar projected into the wound in the cheek, and I extracted it; I also washed out the abscess in the cheek with a solution of carbolic acid, directing the patient to use listerine as a wash. I saw the boy on the third day after this, and decided to open the abscess on the outside of the cheek, the swelling being half as large as an egg. About a teaspoonful of pus was evacuated. The parts healed nicely, the scar being barely perceptible. No doubt in this case the cause of abscess was infection from the roots of the deciduous molar, which was in a state of chronic abscess, the roots being forced into the cheek when struck by the brake-handle.

The fifth case is that of a lady who, four days after having some teeth filled by her dentist, noticed her face on the left side commence to twitch and draw up. In three days from this time she was unable to open her mouth on account of the rigidity of the muscles. Her physician, becoming alarmed, called one of the surgeons of the Massachusetts General Hospital in consultation, who evidently thought she might have tetanus, and stated that she would be a great deal better or a great deal worse by the seventh day. The critical time came, and she was no better and no worse.

At the suggestion of the consulting surgeon that possibly the teeth that were filled might be the cause of the trouble, I was called in to examine. The patient was in bed, and although I knew her by sight I should not have recognized her, her face was so distorted. With a small glass I was able to press out the cheek and make only an examination of the buccal surfaces of the teeth; but as the wisdom-tooth, which was rather small, had an extremely large alloy filling in it, I concluded that the pulp-chamber must have been encroached upon when the cavity was excavated, and, furthermore, the patient had felt but little pain when the tooth was filled. I concluded that the pulp was dead or dying, and advised its being removed. The patient was etherized, and although two hundred and fifty grammes of ether were used, and reflex action seemed to be interfered with, as evidenced by stertorous breathing and loss of sensitiveness of the eye to light or touch, we were unable to overcome the contraction of the muscles of the lower jaw, and it was impossible to open the mouth more than a quarter of an inch. I managed to slip the beak of the forceps into the inside edge of the tooth, and, using the lower tooth for a fulcrum, pried it out of its socket. On removing the filling I found it had been anchored into the pulp-chamber. The remains of the pulp were in a putrescent condition. The patient gradually recovered the use of her jaw, although it was quite stiff for over a year.

The sixth and last case was that of a lady who had been under the physician's care for a number of months for mania. She would have periods of crying, then suddenly jump up and down and yell at the top of her voice. She had lost weight, her digestion was impaired, and she was apparently in consumption. Her teeth being troublesome, her physician requested their removal. Ether was administered, and all her teeth, which were in a terribly broken-down condition, were removed. Inside of two weeks she had recovered her mind, and is now at the end of four months in a good physiological condition.

In closing, I wish to say that I believe we should impress upon the minds of all our medical friends the fact that the intelligent dentist should by right of special education be consulted by them whenever disturbances of the nervous system do not readily yield to general treatment, thereby not only benefiting in many cases their patients, but cementing more closely the relationship between medicine and dentistry.

